# Anastomotic leakage after resection for rectal cancer and recurrence-free survival in relation to postoperative C-reactive protein levels

**DOI:** 10.1007/s00384-024-04766-w

**Published:** 2024-12-02

**Authors:** Anders Gerdin, Jennifer Park, Jenny Häggström, Josefin Segelman, Peter Matthiessen, Marie-Louise Lydrup, Martin Rutegård, on behalf of the RectoLeak Study Group, Östersund Olle Sjöström, Falun Maria Staffan, Växjö Staffan Jangmalm, Växjö Hanna Royson, Uppsala Konstantinos Tsimogiannis, Ersta Kajsa Anderin, Ersta Jonas Nygren, Luleå Jennie Hurtig, Gävle Parisa Golshani

**Affiliations:** 1 Department of Diagnostics and Intervention, Surgery, Umeå University, 901 85 Umeå, Sweden; 2 Department of Surgery, Sahlgrenska University Hospital, SSORG - Scandinavian Surgical Outcomes Research Group, Sahlgrenska Academy, University of Gothenburg, Gothenburg, Sweden; 3 Department of Statistics, Umeå School of Business, Economics and Statistics, Umeå University, Umeå, Sweden; 4 Department of Molecular Medicine and Surgery, Karolinska Institutet, Solna, Sweden; 5 Department of Surgery, Ersta Hospital, Stockholm, Sweden; 6 Department of Surgery, Faculty of Medicine and Health, Örebro University, Örebro, Sweden; 7 Department of Surgery, Skåne University Hospital, Malmö, Lund University, Lund, Sweden

**Keywords:** Anastomotic leakage, Rectal cancer surgery, Mediation analysis

## Abstract

**Background:**

Anastomotic leakage after rectal cancer surgery is linked to reduced survival and higher recurrence rates. While an aggravated inflammatory response may worsen outcomes, few studies have explored the combined effects of leakage and inflammation.

**Methods:**

This is a retrospective multicenter cohort study including patients operated with anterior resection for rectal cancer in Sweden during 2014–2018. Anastomotic leakage within 12 months was exposure and primary outcome was recurrence-free survival. Mediation analysis was performed to evaluate the potential effect of systemic inflammatory response, as measured by the highest postoperative C-reactive protein (CRP) level within 14 days of surgery. Confounders were chosen using a causal diagram.

**Results:**

Some 1036 patients were eligible for analysis, of whom 218 (21%) experienced an anastomotic leakage. At the end of follow-up at a median of 61 months after surgery, recurrence-free survival amounted to 82.6% and 77.8% in the group with and without leakage, respectively. The median highest postoperative CRP value after surgery was higher in the leakage group (219 mg/l), compared with the group without leakage (108 mg/l). Leakage did not lead to worse recurrence-free survival (HR 0.66; 95% CI 0.43–0.94), and there was no apparent effect through postoperative highest CRP (HR 1.12; 95% CI 0.93–1.29).

**Conclusions:**

In conclusion, anastomotic leakage, with its accompanying CRP increase, was not found to be associated with recurrence-free survival after anterior resection for rectal cancer in this patient cohort. Larger, even more detailed studies are needed to further investigate this topic.

**Supplementary Information:**

The online version contains supplementary material available at 10.1007/s00384-024-04766-w.

## Introduction

Anterior resection for rectal cancer is a major procedure, of which one of the more serious complications is anastomotic leakage. In previous population-based studies, anastomotic leakage occurs in 10 to 20% of patients [1, 2]. Established risk factors are male sex, comorbidity, smoking, preoperative radiotherapy, and low anastomosis [3, 4]. Anastomotic leaks are associated with short- and long-term morbidity, and possibly worse overall survival and higher recurrence rates [5].

Whether recurrence is caused by direct tumor spillage, immunosuppression, inflammation, or other mechanisms is not fully understood [6]. Surgical trauma stimulates the release of catecholamines and prostaglandins, which in turn seem to have a pro-metastatic effect and their perioperative inhibition might lower recurrence rates [6, 7]. Some data suggest that patients with symptomatic leakages have higher recurrence rates than patients with asymptomatic leakages [8, 9]. In line with this, the magnitude of the postoperative systemic inflammatory response, as measured by C-reactive protein (CRP), is associated with higher recurrence rates, with or without registered complications [10, 11]. So far, no study has evaluated whether anastomotic leakage has a direct effect on recurrence, or whether the effect is mediated by the inflammatory response, or what magnitude these effects have in isolation or in conjunction to one another.

In this study, we aim to evaluate the relation between anastomotic leakage and recurrence-free survival, considering the postoperative inflammatory response, using formal mediation analysis.

## Method

### Checklist for the reporting of observational studies

The Strengthening the Reporting of Observational Studies in Epidemiology (STROBE) checklist for the reporting of observational studies has been used while writing this article [12].

### Study design

This is a multicenter cohort study with eleven participating centers in Sweden. Patients operated between 2014 and 2018 were identified from theater lists at each hospital. Chart review was conducted at each site and relevant data such as perioperative variables, postoperative highest CRP levels, and postoperative anastomotic leakage were entered into a clinical report form in a REDCap database (Vanderbilt University, TN, USA). The REDCap system is a secure web application for building and managing online surveys and databases [13].

The REDCap database and the Swedish Colorectal Cancer Registry (SCRCR) were linked to ensure that all patients included were operated for rectal cancer and to provide additional information such as demographics, tumor stage and height, and additional perioperative data. The SCRCR was established in 1995 and includes all hospitals in Sweden that operate rectal cancer [14]. The SCRCR has been validated several times with excellent results, demonstrating an average completeness of 99% and overall agreement between registry and re-abstracted variables at 90% between 2008 and 2015 [15]. However, some variables such as anastomotic leakage are known to be underreported [16].

### Inclusion and exclusion criteria

All patients having undergone an anterior resection with anastomosis for rectal cancer were included. Exclusion criteria included patients with disseminated disease at surgery and missing data regarding postoperative CRP, anastomotic leakage, T or N stage, or recurrence.

### Exposure

Any anastomotic leakage within 12 months after the anterior resection was considered the main exposure. Leakage was defined according to the consensus criteria by the International Study Group for Rectal Cancer (ISREC) [17], where any compromised suture or staple line as well as fistulisation to or from the rectum is considered leaks; isolated pelvic abscesses close to the anastomosis are also included. The leaks were subdivided into grade A (leak without need for any treatment, typically asymptomatic), grade B (leak requiring treatment, excluding laparoscopy or laparotomy), and grade C (leak necessitating laparoscopy or laparotomy). Day of leakage was registered as well, where leaks occurring later than 14 days after surgery were assumed to have taken place during the early postoperative period, even though they were diagnosed only later. This assumption allowed the highest postoperative CRP level to be ascribed to initially undetected leaks, which is plausible because many patients, especially those with a defunctioned stoma, are diagnosed with leaks several weeks and even months after surgery [18].

### Mediator

The mediator of interest in this study is the highest CRP level, measured in milligram per liter, during the first 14 postoperative days. This was assumed to reflect the systemic inflammatory response of the patient.

### Outcome

The primary outcome was recurrence-free survival according to chart review. Secondary outcomes were local and distance recurrence as well as overall survival. Local recurrence was defined as any recurrent tumor in the pelvis, where a strong radiological suspicion was deemed sufficient (a positive biopsy or histopathological specimen was not required). Distant recurrence denoted any tumor spread beyond the pelvis not known before surgery.

### Statistical analysis

Frequency tables concerning patient and tumor characteristics were constructed. Continuous variables were described using the median along with the interquartile range (IQR). The association between leak status and recurrence-free as well as overall survival were visualized using Kaplan–Meier curves, where a log rank test was used to test for differences.

Associations between exposure (anastomotic leakage), mediator (postoperative highest CRP), and outcome (5-year recurrence-free survival; 5-year overall survival) were estimated by fitting three separate models. The exposure-mediator relationship was estimated by a linear model regressing exposure on the mediator while the exposure-outcome relationship was estimated by a Cox model regressing exposure on outcome. Baseline confounders were included in both models. These confounders were selected with the use of a causal diagram [19] (Supp Fig. 1), and included age (continuous), sex (male or female), American Society of Anesthesiologists’ (ASA) fitness grade (I, II, or III–IV), current smoking (yes or no), neoadjuvant treatment (none, radiotherapy, or chemoradiotherapy), pathological tumor category (T0, T1–2, T3, or T4), pathological node category (N0, or N1–2), surgical technique (open, or minimally invasive), type of mesorectal excision (total, or partial), defunctioning stoma (yes, or no), blood loss (continuous), year of surgery, and annual hospital volume (continuous). A Cox model including both exposure and confounders was used to estimate the association between mediator and outcome.

A mediation analysis was performed to explore to what extent the effect of anastomotic leakage on survival was mediated by postoperative highest CRP. The direct, indirect, and total effects were estimated using a natural effects Cox model [20]. The direct effect reflects the part of the association between exposure and outcome that is not affected by the mediator, while the indirect effect reflects the part of the association that can be explained by the mediator. As a first step, a parametric survival model, including all confounders and the mediator, was used to model the relationship between exposure and outcome. A duplicate of the exposure variable, exposure*, was then added to the data and a copy of the data was created in which the values in the variable exposure* were set to be counterfactual to the values in the original exposure variable. The data copy was appended to the original data so that the new dataset consisted of twice as many observations as the original data. Then, the first step survival model was used to impute survival times conditional on the exposure* values and observed mediator and confounder values. Finally, a Cox model was fitted to the imputed survival times using the original exposure variable, exposure*, and baseline confounders. The coefficients of exposure* and exposure are estimates of the natural direct and indirect log hazard ratios. This was done 10 times, and the Cox model fits were pooled using Rubin’s rules [21]. Confidence intervals of the natural effects were based on 1000 bootstrap samples [22].

A dataset where the missing values were imputed by multiple imputation by chained equations was used in the analyses [23].

The above analyses were subsequently repeated for a subset of the cohort, where grade A and B leaks were excluded, retaining only patients without anastomotic leakage and a grade C leak group; this was done to evaluate whether severe leakage imparts a more pronounced effect, and potentially also to alleviate diagnostic bias between centers as such leaks were considered straightforward to detect. Finally, a sensitivity analysis was conducted to evaluate whether chronic inflammation, using the proxy of leakage with and without the anastomosis in situ as exposure, would impact recurrence-free survival.

In a post hoc analysis sensitivity analysis, propensity score matching was performed. The propensity score was estimated using a logistic regression model that included all baseline confounders. Pairs of exposed and unexposed patients were matched on the logit of the propensity score using a caliper of width equal to 0.2 of the standard deviation of the logit of the propensity score. In the matched sample, a Cox model was used to estimate the impact of any anastomotic leakage on 5-year recurrence-free survival, with a robust variance estimator used to account for the clustering within matched sets.

All analyses were performed using R 4.2.3 statistical software [24]. The R-package mice [25] was used for imputation.

## Results

### Patients

**Fig. 1:**
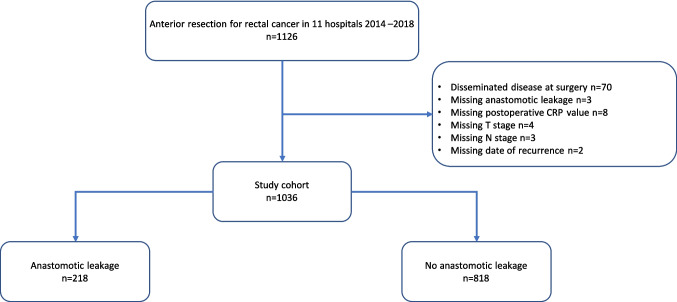
Study flowchart

Between 2014 and 2018, a total of 1126 patients at 11 hospitals were operated with anterior resection for rectal cancer and entered into the REDCap database. Of these, 90 patients were excluded (Fig. 1), leaving a total of 1036 patients in the final analysis. These patients were followed up at a median of 1865 days (61 months) after surgery (IQR 1377–2342). During the same period, a total of 1207 patients were registered in the SCRCR from the participating hospitals. The cohort in this study thus represents 86% of the potentially eligible population from the participating hospitals. Moreover, the present cohort comprised 31% (1126/3325) of all the anterior resections registered in the SCRCR nationwide during the study period.

Baseline clinical variables and characteristics are described in Table 1. The anastomotic leakage rate was 21.0%; of these, grade A was 14.2%, grade B amounted to 56.4%, and grade C constituted 29.4%. The median postoperative highest CRP value after surgery was higher in the leakage group (median 218.5 mg/l), compared with the group without leakage (median 108 mg/l). Patients with a leakage were more often male, with a higher ASA grade, and active smokers. Neoadjuvant therapy and total mesorectal excision were also more common in the leakage group. There were no notable differences between groups regarding age, BMI, tumor staging, minimally invasive surgery, and intraoperative bleeding.

**Table 1:** Baseline characteristics derived from full sample, *N* = 1036, incl. missing values

	No leak (*N* = 818)	Leak (*N* = 218)	Overall (*N* = 1036)
Postop highest CRP value (mg/L)			
Median (IQR)	108.0 (59.3; 169.0)	218.5 (140.0; 308.5)	124.0 (69.0; 204.3)
Age (years)			
Median (IQR)	67 (60; 73)	66 (59; 73)	67 (60; 73)
Missing	1 (0.1%)	0 (0%)	1 (0.1%)
Sex			
Female	345 (42.2%)	69 (29.4%)	409 (39.5%)
Male	473 (57.8%)	154 (70.6%)	627 (60.5%)
ASA			
I	186 (22.7%)	47 (21.6%)	233 (22.5%)
II	491 (60.0%)	122 (56.0%)	613 (59.2%)
III–V	135 (16.5%)	46 (21.1%)	181 (17.5%)
Missing	6 (0.7%)	3 (1.4%)	9 (0.9%)
Smoker			
No	706 (86.3%)	177 (81.2%)	883 (85.2%)
Yes	50 (6.1%)	19 (8.7%)	69 (6.7%)
Missing	62 (7.6%)	22 (10.1%)	84 (8.1%)
Body mass index (kg/m^2^)			
Median (IQR)	25.5 (23.2; 28.3)	25.9 (23.8; 29.0)	25.6 (23.4; 28.4)
Missing	15 (1.8%)	3 (1.4%)	18 (1.7%)
Tumor height (cm)			
Median (IQR)	10 (9; 13)	10 (8; 12)	10 (9; 12)
Missing	2 (0.2%)	3 (1.4%)	5 (0.5%)
Neoadjuvant therapy			
No	453 (55.4%)	74 (33.9%)	527 (50.9%)
Radiotherapy	256 (31.6%)	102 (46.8%)	358 (34.6%)
Radio-chemotherapy	109 (13.3%)	42 (19.3%)	151 (14.6%)
Pathological tumor category			
(y)pT0	15 (1.8%)	3 (1.4%)	18 (1.7%)
(y)pT1-2	331 (40.5%)	76 (34.9%)	407 (39.3%)
(y)pT3	414 (50.6%)	127 (58.3%)	541 (52.2%)
(y)pT4	48 (5.9%)	11 (5.0%)	59 (5.7%)
Missing	10 (1.2%)	1 (0.5%)	11 (1.1%)
Pathological node category			
(y)pN0	508 (62.1%)	142 (65.1%)	650 (62.7%)
(y)pN1-2	297 (36.3%)	75 (34.4%)	372 (35.9%)
Missing	13 (1.6%)	1 (0.5%)	14 (1.4%)
Operation year			
Median (IQR)	2016 (2015; 2017)	2016(2015; 2017)	2016 (2015; 2017)
Hospital volume (annual)			
Median (IQR)	20.0 (17.0; 35.8)	21.4 (15.4; 46.2)	20.0 (17.0; 35.8)
Surgical technique			
Open	363 (44.4%)	107 (49.1%)	470 (45.4%)
Minimally invasive	454 (55.5%)	111 (50.9%)	565 (54.5%)
Missing	1 (0.1%)	0 (0%)	1 (0.1%)
Type of mesorectal excision			
Total	617 (75.4%)	200 (91.7%)	817 (78.9%)
Partial	201 (24.6%)	18 (8.3%)	219 (21.1%)
Defunctioning stoma			
No	166 (20.3%)	27 (12.4%)	193 (18.6%)
Yes	652 (79.7%)	191 (87.6%)	843 (81.4%)
Perioperative bleeding (ml)			
Median (IQR)	150 (50; 400)	200 (100; 500)	150 (50; 400)
Missing	19 (2.3%)	3 (1.4%)	22 (2.1%)

*CRP* C-reactive protein, *ASA* American Society of Anesthesiologists, *IQR* interquartile range

### Mortality and recurrence rate

**Fig. 2:**
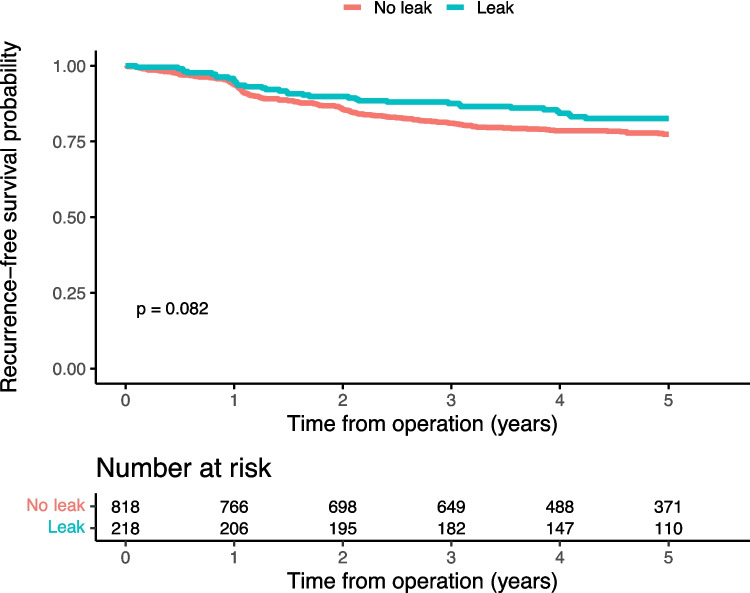
Kaplan–Meier curves with log-rank test on, by leak status, recurrence-free 5-year survival

**Fig. 3:**
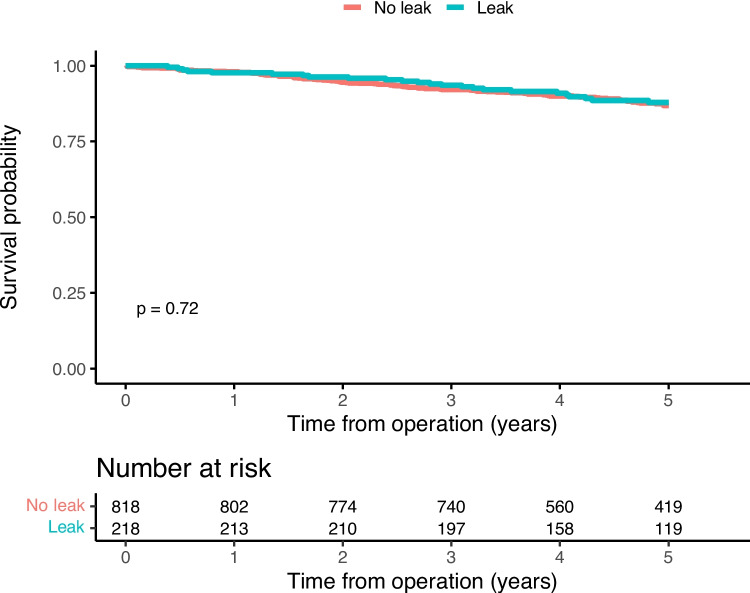
Kaplan–Meier curves with log-rank test, by leak status, overall 5-year survival

**Fig. 4:**
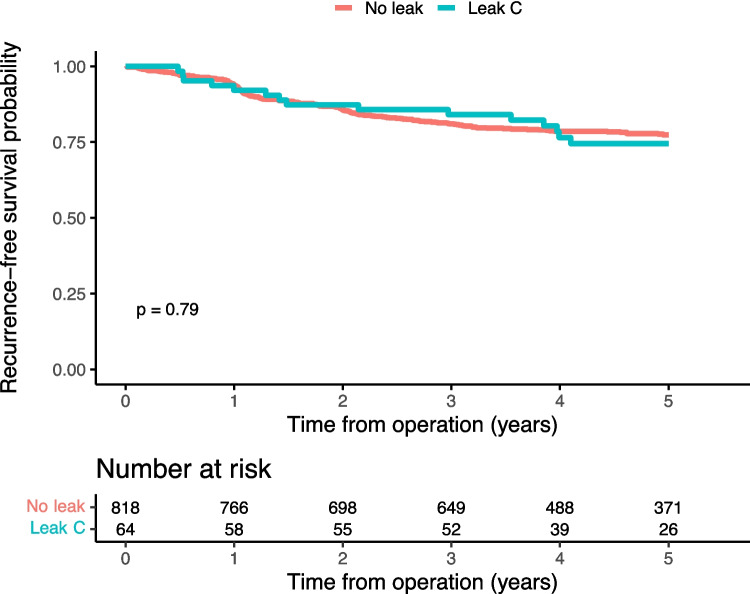
Kaplan–Meier curves with log-rank test, by leak status, recurrence-free 5-year survival

**Fig. 5:**
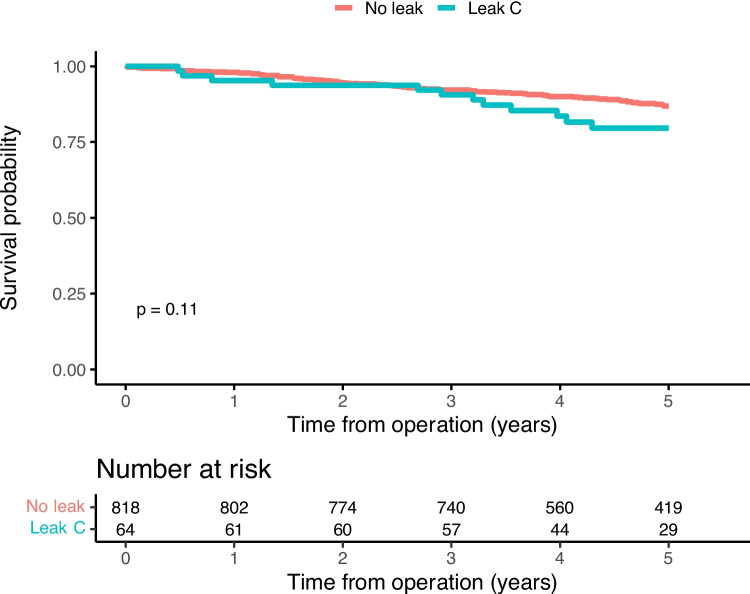
Kaplan–Meier curves with log-rank test, by leak status, overall 5-year survival

At end of follow-up, mortality had occurred in 11.5% and 12.6% of patients with and without anastomotic leakage, respectively. Local recurrence occurred in 2.6% of the patients without leakage, while this was observed in 1.4% of the patients with leakage. In the group without leakage, distant recurrence was observed in 15.6% of the patients, compared to 11.9% of the patients with leakage. The main outcome recurrence-free survival amounted to 82.6% in the group having sustained anastomotic leakage whereas the corresponding figure was 77.8% in the group without leakage (Fig. 2), while overall survival was similar in both groups (Fig. 3). Considering different leak grades, there were differences in recurrence-free (Supp Fig. 2) and overall survival (Supp Fig. 3), suggesting that grade A and B leaks were associated with outcomes similar to or better than those without a leakage, while patients with a grade C leakage had similar recurrence-free survival (Fig. 4) but possibly worse overall survival than patients without anastomotic leakage (Fig. 5). In the sensitivity analysis, there was no reduction in recurrence-free survival in patients with leakage and an anastomosis in situ, compared to those with leakage but without a remaining anastomosis, or to those without a leakage (data not shown).

### Mediation analysis

When modeling the impact of leakage (the exposure) on the highest CRP concentration seen postoperatively (the mediator), an increase of 98.79 mg/l (95% CI: 85.07–112.51) was seen in patients with leakage compared to those without. The corresponding influence from leakage on time to recurrence or death (the outcome) was estimated with an HR of 0.65 (95% CI 0.45–0.94). At the same time, the postoperative CRP influence on time to recurrence or death amounted to an HR of 1.00 (95% CI 1.00–1.00). This information is summarized in Table 2.

**Table 2:** Associations between anastomotic leakage (exposure), postoperative highest CRP (mediator), and 5-year recurrence-free survival (outcome)

		95% CI		
Model	Coef/HR	Lower limit	Upper limit	*p*-value
Exposure (leak)-mediator (CRP)	98.79	85.07	112.51	< 0.001
Exposure (leak)-outcome (recurrence-free survival)	0.65	0.45	0.94	0.022
Mediator (CRP)-outcome (recurrence-free survival)	1.00	1.00	1.00	0.263

The results are based on linear (regressing exposure on mediator) and Cox models (regressing exposure on outcome; regressing mediator on outcome). All models include baseline confounders. In the mediator-outcome model, the exposure is included in addition to baseline confounders

The mediation analysis based on the natural effects Cox model resulted in an estimated total HR of 0.66 (95% CI 0.43–0.94), indicating a decrease in recurrence or death in the leakage group. The natural indirect effect, mediation through CRP increase, amounted to an HR of 1.12 (95% CI 0.93–1.29), effectively suggesting no discernible impact on the outcome. The natural direct effect, in turn, consisted of an HR of 0.59 (95% CI 0.38–0.86), implying that the total effect comprised the direct effect to a substantial degree (Table 3).

**Table 3:** Effect of anastomotic leakage on 5-year recurrence-free survival mediated by postoperative highest CRP

Effect	HR	95% CI	
		Lower limit	Upper limit
Natural direct effect	0.590	0.380	0.855
Natural indirect effect	1.123	0.932	1.293
Total effect	0.663	0.428	0.941

The results are based on a natural effects Cox model conditional on baseline confounders. Effects are hazard ratios (HRs) for recurrence or death

When considering death only as the outcome, the patterns described above were similar, while the point estimates for the natural direct and total effects were closer to the null, with wide confidence intervals (Tables 4 and 5).

**Table 4:** Associations between anastomotic leakage (exposure), postoperative highest CRP (mediator), and 5-year overall survival (outcome)

		95% CI		
Model	Coef/OR/HR	Lower limit	Upper limit	*p*-value
Exposure (leak)-mediator (CRP)	98.79	85.07	112.51	< 0.001
Exposure (leak)-outcome (overall survival)	0.82	0.51	1.32	0.415
Mediator (CRP)-outcome (overall survival)	1.00	1.00	1.00	0.056

The results are based on linear (regressing exposure on mediator) and Cox models (regressing exposure on outcome; regressing mediator on outcome). All models include baseline confounders. In the mediator-outcome model, the exposure is included in addition to baseline confounders

**Table 5:** Effect of anastomotic leakage on 5-year overall survival mediated by postoperative highest CRP

Effect	HR	95% CI	
		Lower limit	Upper limit
Natural direct effect	0.721	0.384	1.237
Natural indirect effect	1.139	0.958	1.477
Total effect	0.822	0.454	1.406

The results are based on a natural effects Cox model conditional on baseline confounders. Effects are hazard ratios (HRs) for death

In the sensitivity analysis of 882 patients, considering only grade C leakage versus no leakage, these groups had a mortality rate of 20.3% and 12.6%, respectively, with no appreciable differences in recurrence rates. When repeating the mediation analysis for this restricted data set, the total effect regarding recurrence or death was estimated at an HR of 1.07 (95% CI 0.56–1.78), while the decomposed effects were also close to unity (Supp Table 1). When considering death only as the outcome (Supp Table 2), the total effect HR was 1.45 (95% CI 0.64–2.72), reflected by an indirect effect at an HR of 1.37 (95% CI 0.93–1.83).

The post hoc propensity score matching produced a balanced set of covariates, with all absolute mean differences below 0.10. Recurrence-free survival amounted to 83.9% in the group having sustained anastomotic leakage, whereas the corresponding figure was 75.6% in the group without leakage. The estimated hazard ratio was 0.64 (95% CI 0.42–0.97).

## Discussion

In this large retrospective cohort study of patients operated with anterior resection for rectal cancer, there was no evidence of an effect of anastomotic leakage on recurrence-free survival. Moreover, postoperative systemic inflammation as measured by the highest CRP value after surgery did not seem to influence risk of recurrence or death. In addition, there was also no discernible effect when evaluating grade C leaks only.

The study cohort includes patients operated in a modern time period from both university and county hospitals, contributing to generalizability. The anastomotic leakage rate was high, but similar to other recent multicentre studies with a longer follow-up [2]. Risk factors for anastomotic leakage were distributed as anticipated, with a preponderance of men, smokers, comorbidity, neoadjuvant treatment, and total mesorectal excision in the leakage group [3]. While partly based on registry data, an advantage of the present study is the thorough chart review, enabling capture of all anastomotic leaks long after surgery, although bearing in mind that data were retrieved retrospectively. The sample size is quite large, but subgroup analyses concerning leak grades were nevertheless difficult to conduct. Modern statistical methods have been employed, including multiple imputation for missing values and careful adjustment for confounding. There are, however, limitations in this study. Firstly, residual confounding cannot be ruled out in an observational setting, not the least due to information bias when collecting retrospective data. Secondly, using a single CRP value in the immediate postoperative period might be an oversimplification of a complex and dynamic process; a more detailed data capture could have rendered more information of use, while potentially making the data collection too cumbersome to execute. Thirdly, some patients in the later part of the study had short follow-up times, possibly leading to artificially low long-term outcomes.

Meta-analysis data concerning anastomotic leakage after anterior resection for rectal cancer generally demonstrate a reduced overall survival, while the association to distant recurrence is less clear [5, 26]. Locoregional recurrence, however, seems to be more affected, as also suggested by long-term follow-up in the COLOR II study [27]. These observations are, nevertheless, somewhat hampered by the lack of uniform definition and grading of anastomotic leakage, potentially making attempts of meta-analysis difficult. When evaluating, e.g., asymptomatic grade A leaks, no impact on survival has been noted [8, 9], consistent with the present study. However, the abovementioned studies and others have found reduced recurrence-free survival in symptomatic leaks (grade B/C), in contrast to our findings; these discrepancies might be explained by differences in leak definition, data capture, and leakage treatment, or even by the fact that the current study is based on a more contemporary cohort. Nevertheless, a recent prospective multicenter study based on trial data could not show any association between anastomotic leakage and recurrence or overall survival [28]. Interestingly, we were also unable to reproduce findings from other studies showing that a higher postoperative inflammatory response could be associated to worse overall survival or recurrence [10, 11]. One explanation for this, despite the theoretical soundness of the connection between inflammation and recurrence, is that there is no true effect at hand. Alternatively, modern healthcare might have become successively better at selecting patients for the right kind of surgery (e.g., not offering an anastomosis for frail patients) or managing complications; while such reasoning might explain the lack of effect on mortality, it is more difficult to explain the lack of influence on recurrence rates. According to a recent study [29], patients with elevated CRP levels after the first postoperative month have lower overall and colorectal cancer-specific survival rates. This would suggest that chronic inflammation may be the culprit, rather than the acutely elevated CRP levels measured in the present study. However, this hypothesis was tested to some degree herein, without any evidence of worse recurrence-free survival in those with an anastomosis in situ despite an anastomotic leakage, potentially also with a chronic inflammation.

In conclusion, anastomotic leakage, with its accompanying CRP increase, might not be a driver for recurrence and long-term death after anterior resection for rectal cancer. Larger, even more detailed studies, comprising postoperative CRP trajectories or other measures of systemic inflammation as well as more events with severe anastomotic leaks, are certainly needed to investigate this topic further.

## Supplementary Information

Below is the link to the electronic supplementary material.

Supplementary file1 (DOCX 592 KB)

Supplementary file2 (DOCX 29.4 KB)

## Data Availability

Upon reasonable request, data and methodology can be shared. This also applies to the registry-based data used in the current study, while access to such data might be subject to external review by the Swedish Colorectal Cancer Registry steering committee.

## Declarations

### Permission to reproduce material from other sources

No material from other sources was included in this article.
