# Supplementary material for: Anastomotic leakage after resection for rectal cancer and recurrence-free survival in relation to postoperative C-reactive protein levels
Source: Int J Colorectal Dis. 2024 Dec 2;39(1):193. doi: 10.1007/s00384-024-04766-w (PMC11611975; doi:10.1007/s00384-024-04766-w)
Supplement: Supplementary file 1 — Supplementary file1 (DOCX 592 KB) [file 384_2024_4766_MOESM1_ESM.docx]

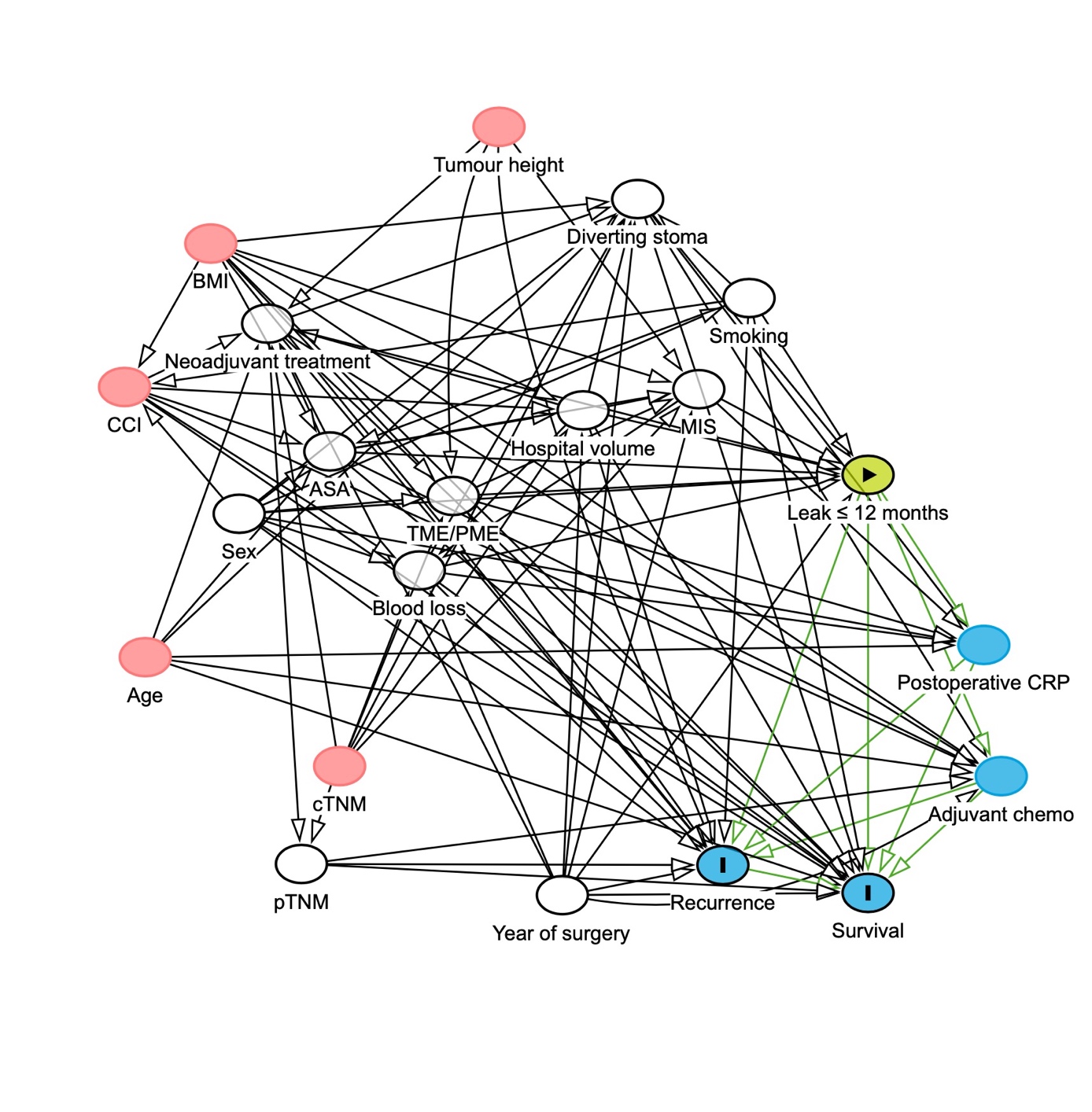


**Supp Figure 1.** Directed acyclic graph. The green circle indicates the exposure. The blue circumscribed circle indicates outcomes. Red or white circles indicate ancestors of the exposure and the outcomes, where white circles denote adjusted confounders sufficient to estimate the total effect. Blue uncircumscribed circles indicate mediators on the causal pathway between the exposure and the outcomes.

**Supp Figure 2.** Kaplan-Meier curves with log-rank test, by leak status, recurrence-free 5-year survival.

**Supp Figure 3.** Kaplan-Meier curves with log-rank test, by leak status, overall 5-year survival.
