# Supplementary material for: Anastomotic leakage after resection for rectal cancer and recurrence-free survival in relation to postoperative C-reactive protein levels
Source: Int J Colorectal Dis. 2024 Dec 2;39(1):193. doi: 10.1007/s00384-024-04766-w (PMC11611975; doi:10.1007/s00384-024-04766-w)
Supplement: Supplementary file 2 — Supplementary file2 (DOCX 29.4 KB) [file 384_2024_4766_MOESM2_ESM.docx]

**Supp Table 1.** Leak C vs. no leakage. Recurrence or death, survival mediation analysis (Jenny’s code). Results from estimating a natural effects model (Lange et al, 2017).

| **Effect** | **HR** | **95% CI** | |
| --- | --- | --- | --- |
|  |  | **Lower limit** | **Upper limit** |
| *5-year recurrence-free survival* |  |  |  |
| Natural direct effect | 0.959 | 0.491 | 1.711 |
| Natural indirect effect | 1.118 | 0.831 | 1.427 |
| Total effect | 1.073 | 0.560 | 1.780 |

**Supp Table 2**. Leak C vs. no leakage. Death, survival mediation analysis (Jenny’s code). Results from estimating a natural effects model (Lange et al, 2017).

| **Effect** | **HR** | **95% CI** | |
| --- | --- | --- | --- |
|  |  | **Lower limit** | **Upper limit** |
| *5-year overall survival* |  |  |  |
| Natural direct effect | 1.062 | 0.495 | 2.140 |
| Natural indirect effect | 1.367 | 0.934 | 1.832 |
| Total effect | 1.452 | 0.638 | 2.715 |
